# Regulation of gene expression by photosynthetic signals triggered through modified CO_2 _availability

**DOI:** 10.1186/1471-2229-6-15

**Published:** 2006-08-17

**Authors:** Dennis Wormuth, Margarete Baier, Andrea Kandlbinder, Renate Scheibe, Wolfram Hartung, Karl-Josef Dietz

**Affiliations:** 1 Biochemistry and Physiology of Plants, Bielefeld University – W5, 33501 Bielefeld, Germany; 2 Plant Physiology, University of Osnabrück, FB 5, 49069 Osnabrück, Germany; 3 Molecular Plant Physiology and Biophysics, Julius von Sachs-Institut für Biowissenschaften, 97082 Würzburg, Germany

## Abstract

**Background:**

To coordinate metabolite fluxes and energy availability, plants adjust metabolism and gene expression to environmental changes through employment of interacting signalling pathways.

**Results:**

Comparing the response of Arabidopsis wild-type plants with that of the mutants *adg1*, *pgr1 *and *vtc1 *upon altered CO_2_-availability, the regulatory role of the cellular energy status, photosynthetic electron transport, the redox state and concentration of ascorbate and glutathione and the assimilatory force was analyzed in relation to the transcript abundance of stress-responsive nuclear encoded genes and psaA and psbA encoding the reaction centre proteins of photosystem I and II, respectively. Transcript abundance of Bap1, Stp1, psaA and psaB was coupled with seven metabolic parameters. Especially for psaA and psaB, the complex analysis demonstrated that the assumed PQ-dependent redox control is subordinate to signals linked to the relative availability of 3-PGA and DHAP, which define the assimilatory force. For the transcripts of sAPx and Csd2 high correlations with the calculated redox state of NADPH were observed in *pgr1*, but not in wild-type, suggesting that in wild-type plants signals depending on thylakoid acidification overlay a predominant redox-signal. Strongest correlation with the redox state of ascorbate was observed for 2CPA, whose transcript abundance regulation however was almost insensitive to the ascorbate content demonstrating dominance of redox regulation over metabolite sensing.

**Conclusion:**

In the mutants, signalling pathways are partially uncoupled, demonstrating dominance of metabolic control of photoreaction centre expression over sensing the redox state of the PQ-pool. The balance between the cellular redox poise and the energy signature regulates sAPx and Csd2 transcript abundance, while 2CPA expression is primarily redox-controlled.

## Background

Photosynthesis provides plant cells with assimilates, reducing power and ATP. To coordinate their supply and demand, plants respond to environmental changes on time scales ranging from milliseconds to days. In addition to biochemical regulation of enzyme activities, control of gene expression is essential for long-term adjustment of metabolic capacities. However, the photosynthetic electron transport chain and chloroplast metabolism have evolved as patchworks composed of nuclear and plastid-encoded proteins and depend on coordination of gene expression between compartments. In recent years, physiological and mutant approaches addressed the regulatory function of metabolite signals [1], energy status [2] and chloroplast redox signals [3]. For example, carbohydrates induce expression of genes involved in starch biosynthesis and suppress those involved in starch degradation and carbon assimilation [4]. In parallel, in the plastids, expression of e.g. core subunits of the photoreaction centres and the large subunit of RuBisCO are suppressed either by co-regulation of transcription or epistatically. Photosynthates further inhibit the Calvin-Cycle biochemically [5] and decrease the consumption of NADPH and ATP, which results in an increased reduction state of the NAD(P)-system and a higher phosphorylation state of adenylates [6,7]. The lack of NADP^+ ^as electron acceptor and the generation of a high trans-thylakoid ΔpH by decreased photophosphorylation reduce the carriers of intersystem electron transport, like plastoquinone pool (PQ), and stimulate ROS formation [8-10]. PQH_2 _and ROS are redox signals controlling nuclear and plastid gene expression [11,12]. The combination of decreased NADP^+ ^regeneration and high thylakoid acidification promotes the violaxanthin cycle, which can support biosynthesis of the plant hormone abscisic acid [13] if ascorbate availability is limiting [14].

The plant signalling networks evolved in the context of a strong interference of photosynthates and redox and energy signals. Therefore, in *wt *distinction of particular signals is often difficult. Here, for differentiation, gene expression regulation was analyzed in *wt*, in the thylakoid acidification mutant *pgr1 *[15], in the starch-biosynthetic mutant *adg1 *[16] and in the ascorbate-deficient mutant *vtc1 *[17] in response to the CO_2 _availability.

*pgr1 *is mutated in the Rieske subunit of the cytochrome b_6_f complex (PetC; [15]) that is involved in electron and proton transfer processes between photosystem II and I as a plastohydroquinol-plastocyanin reductase. Due to a shift in the pK or the redox potential of the Rieske protein, thylakoid lumen acidification is restricted in the mutant to pH 6 even in high light [18]. Consequently, the mutant has altered capacities for ADP photophosphorylation [19] and PsbS protonation [20], and may be limited in violaxanthin de-epoxidation [21] and cyclic electron transport [22]. In *pgr1*, high light intensities increase the electron pressure to the PQ pool and release the electron pressure downstream of the cytb_6_f complex. *Pgr1 *was selected in this communication for its limitation in membrane energization and photosynthetic electron transport.

*adg1 *carries a mutation in the small subunit of ADP-glucose pyrophosphorylase [16]. It was selected for its altered carbohydrate metabolism. In contrast to *pgr1*, in *adg1 *photosynthetic electron transport is affected only indirectly by carbohydrate-induced feedback inhibition [23].

The third selected mutant, *vtc1*, carries a point mutation in GDP-mannose pyrophosphorylase [17], which catalyzes precursor formation in the biosynthesis of the low-molecular weight antioxidant ascorbate. *vtc1 *was included in this analysis to investigate the importance of ascorbate availability and ascorbate-related redox processes on nuclear and plastid gene expression.

Arabidopsis *wt *and the mutants were compared under conditions of limiting, ambient and saturating CO_2 _concentrations. Below the CO_2 _compensation point, RuBisCO preferentially catalyzes oxygenation of ribulose-1,5-bisphosphate and activates photorespiration, which triggers an extra demand for ATP. Per reaction cycle, chloroplastic NADPH consumption is low under photorespiratory as compared to assimilatory conditions. In addition, at very low CO_2 _concentrations, depletion of the carbohydrate pool may suppress photorespiration and limit photoprotection. Concomitantly, the ROS load increases due to high peroxisomal H_2_O_2 _production and chloroplast superoxide generation. At ambient conditions, 30 – 40% of the carbon fluxes diverts to photorespiratory CO_2 _release, while saturating CO_2 _suppresses photorespiration and the requirement for ATP to NADPH approaches a ratio of 3:2. In parallel, energization and reduction state of the cells are decreased [24].

The hypothesis of the work was that the combination of working with defined mutants and altering CO_2 _availability for modulation of photosynthesis allows addressing the question of how selected nuclear and plastidic genes are regulated in *Arabidopsis *in response to redox, metabolite and energy signals.

## Results

The work aimed at differentiating signals involved in the control of gene expression. To this end *Arabidopsis wt *and mutants defective in chloroplast starch biosynthesis (*adg1*; [16]), thylakoid acidification (*pgr1*; [15]) and the biosynthesis of the major low-molecular-weight antioxidant ascorbate (*vtc1*; [17]) were compared in relation to their metabolite patterns, photosynthetic performance and transcript amount regulation. Following growth at ambient conditions, the CO_2 _availability was decreased for 6 h to levels below the CO_2 _compensation point or increased to saturation of RuBisCO to establish contrasting conditions for photosynthesis with high and low acceptor availability and concomitant high and low reduction pressure.

## Metabolic regulation

### The energy status

In *wt *during the 6 h fumigation period the ADP content increased 1.5-2-fold irrespective of the treatment (Table 1). In parallel, the ATP accumulated only in 0 ppm CO_2_, but decreased in 350 and 2000 ppm CO_2_. The ATP/ADP ratio was nearly unchanged around 6 in 0 ppm, but decreased to 2.2 and 1.8, respectively, in 350 and 2000 ppm CO_2 _(Table 1). Mutant metabolism resulted in specific modifications of the *wt*-pattern:

In *adg1*, ATP and ADP accumulated 2.2- and 5.5-fold in 0 ppm CO_2 _and 1.3- and 1.9-fold in 350 ppm CO_2 _indicating an increase in the total adenylate concentration, but a decrease in the ATP/ADP ratio. In 2000 ppm CO_2 _the ATP content increased insignificantly, while the ADP content decreased resulting in an ATP/ADP ratio similar to the initial values obtained before the treatment (Table 1).

The ATP content of *vtc1 *slightly but steadily decreased with increasing CO_2 _availability, while the ADP content increased from the lowest levels observed prior to the fumigation experiment 6.2-fold in 0 ppm CO_2_, 2.8-fold in 350 ppm CO_2 _and 3.3-fold in 2000 ppm CO_2 _(Table 1). The high ATP and especially the low ADP contents after 6 h resulted in a strong decrease in the ATP/ADP-ratio from 9.8 to around 2 (Table 1).

*pgr1 *is limited in thylakoid acidification [18]. Like in *vtc1*, the ATP/ADP ratio decreased to values around two during the experiment independent of the CO_2 _concentration applied (Table 1). However, the relative decrease was much less in *pgr1 *due to an already much lower initial ATP/ADP ratio (*vtc1*: 9.8; *pgr1*: 2.9). In addition to the generally low ATP/ADP ratio, the total adenylate concentration was also low in *pgr1 *suggesting coupling between adenylate biosynthesis and ADP-phosphorylation efficiency. The ADP contents were similar to that in *wt *and did not increase in 0 ppm like in *vtc1 *and *adg1 *(Table 1), while the ATP content slightly decreased demonstrating a mutation-specific difference in regulation of the adenylate concentration in parallel to the ATP/ADP ratio (Table 1).

### Assimilatory force and the reduction state of the NADP system

The assimilatory force F_A_, i.e. the product of the phosphorylation potential [ATP]/[ADP] [P_i_] and the ratio of [NADPH]/[NADP^+^], was calculated from the DHAP/3-PGA ratio (Table 1) as introduced in [24]. F_A _indicates the energization of metabolism by photosynthetic light reactions as compared to the consumptive demand. The relationship was originally defined for chloroplasts. Due to energy and metabolite coupling it also tentatively describes the energy status of leaves [25]. F_A _was increased in *wt *and all mutants during 6 h in CO_2_-free air compared to the values at onset of fumigation (*wt*: 180%; *adg1*: 352%; *pgr1 *180%; *vtc1*: 347%) (Table 1) indicating a high reduction state of the NADP-system (*wt*: 148%; *adg1*: 385%; *pgr1 *194%;*vtc1*: 326% increase compared to the onset of illumination) (Table 1). At high CO_2_, the [NADPH]/[NADP^+^]_calc _ratios of the mutants were little increased in *wt *(175%), *adg1 *(155%) and *pgr1 *(123%), but elevated in *vtc1 *(366%) compared to the values at onset of fumigation (Table 1). The calculated reduction states of NADP at high CO_2 _were increased in *wt *(241%), *adg1 *(152%) and *pgr1 *(153%), but hardly changed in *vtc1 *(108%) relative to ambient conditions (Table 1).

The strong decline in the 3-PGA content in CO_2_-free air indicated an increased assimilatory force in low CO_2 _(*wt*: 685%; *adg1*: 511%; *pgr1*: 301%; *vtc1*: 326% relative to ambient air). 3-PGA contents slightly increased in 350 ppm CO_2 _during the 6 h of treatment, remained unchanged in *wt *in 2000 ppm CO_2 _and decreased in the mutants.

### Antioxidant protection

Antioxidant enzymes and low molecular weight redox metabolites constitute the antioxidant defence system of the plants. Modifications of antioxidant enzyme activities often reflect general changes in the redox status and in ROS generation of the tissue, or compensatory responses to specific redox changes [26].

#### Ascorbate contents and redox states

**Figure 1:**
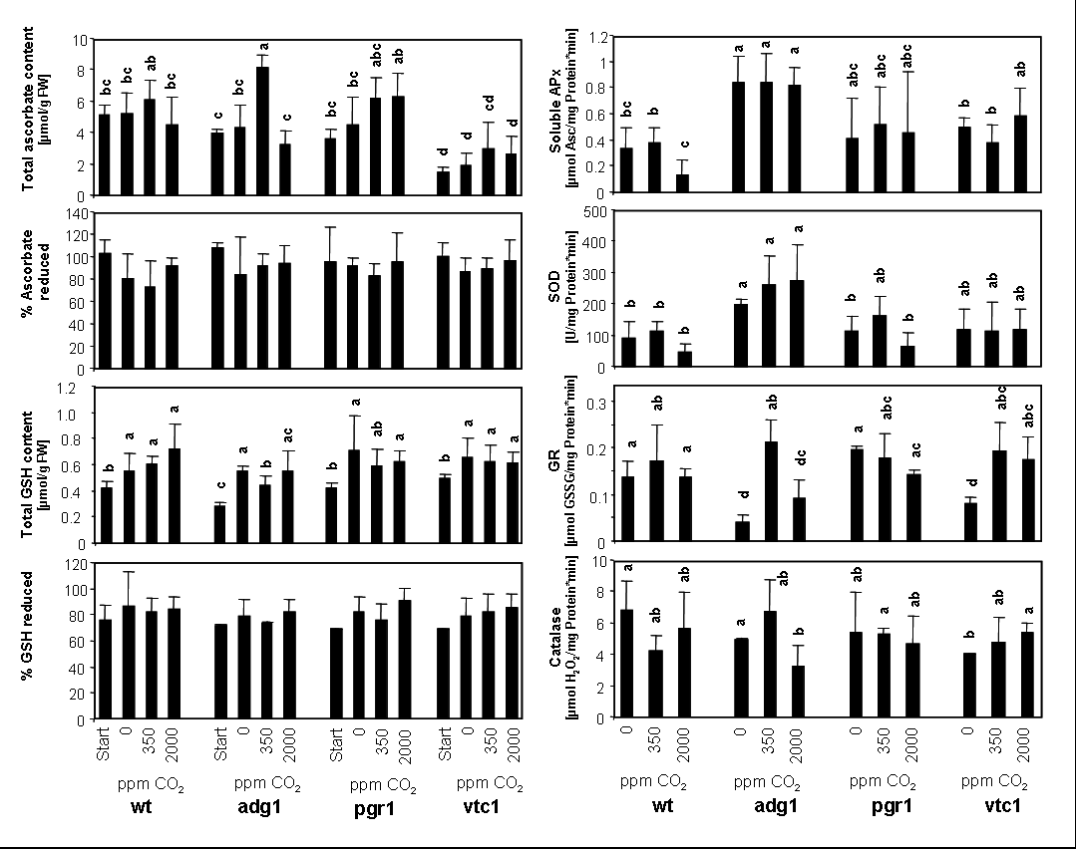
**Redox stabilization and antioxidant defense in response to differing CO_2 _availability: Content of total antioxidant amount and reduction status **(ascorbate and glutathione) and activities of selected antioxidative enzymes: total soluble **ascorbate peroxidases **(APx), total soluble **superoxide dismutases **(SOD), **glutathione reductase **(GR) and **catalases **(CAT) in *wt, adg1, pgr1 *and *vtc1 *under different CO_2 _regimes (0 ppm, 350 ppm and 2000 ppm CO_2_) measured in aqueous extracts. The data are means of n = 4–6, ± SE from at least 4 different experiments.

At the beginning of the fumigation period, the ascorbate content was lowest in *vtc1 *with 1.44 ± 0.31 μmol Asc/g FW reflecting the mutational defect in ascorbate biosynthesis [17] (Fig. 1). The highest ascorbate levels were observed in *wt*, and intermediate contents in *adg1 *and *pgr1*. In *wt *the ascorbate content increased approximately 1.3-fold in ambient air, but hardly changed in low and high CO_2_. In *adg1*, starting with a lower content than *wt*, ascorbate levels increased to high values at 350 ppm and were unchanged in 0 and 2000 ppm CO_2_. In *vtc1*, with its low ascorbate contents due to the mutation in one of the main ascorbate biosynthetic enzymes, ascorbate increased 1.3-fold in CO_2_-free air and 2.1- and 1.8-fold in 350 and 2000 ppm CO_2_. It maximally reached 50% of the *wt *level in 350 ppm CO_2_. In *pgr1*, the ascorbate content increased to 4.5 ± 0.75 μmol Asc/g FW in 0 ppm and to similar levels around 6 μmol Asc/g FW in 350 ppm and 2000 ppm CO_2_. The CO_2 _availability affected the redox state of ascorbate to a minor extent. From an almost fully reduced level at the beginning of the fumigation period, the mean values after 6 h fumigation indicate slightly higher oxidation levels (72 – 91%) in 0 ppm and 350 ppm CO_2 _than in 2000 ppm CO_2 _(Figure 1).

#### Glutathione contents and redox states

Compensating the low ascorbate content, the glutathione content was highest in *vtc1 *at the beginning of the fumigation period. Lowest glutathione contents were observed in *adg1*, while the glutathione content was similar to *wt *in *pgr1*. During the 6 h fumigation the glutathione content increased in all samples to similar levels and redox states (Figure 1). Although not significantly different, the mean values indicate a trend towards slightly higher reduction at the end of the fumigation period (Figure 1).

#### Activities of antioxidant enzymes

Compared to *wt*, the three mutants revealed increased ascorbate peroxidase activities in high and low CO_2_. APx activity of *adg1 *was twice that observed in *wt *at ambient CO_2 _concentrations (Figure 1). In parallel, the SOD activity was 1.5-fold induced in *adg1*. Like for APx the CO_2 _availability scarcely affected SOD activity of *vtc1*. SOD and APx activities were only slightly higher in *pgr1 *than in *wt*, while that mutation increased glutathione reductase activity in 0 and 350 ppm CO_2 _(Figure 1). Surprisingly, SOD and GR activities were lower in 0 than 350 ppm CO_2 _(Figure 1).

### Contents of soluble and hydrolysable sugars

**Figure 2:**
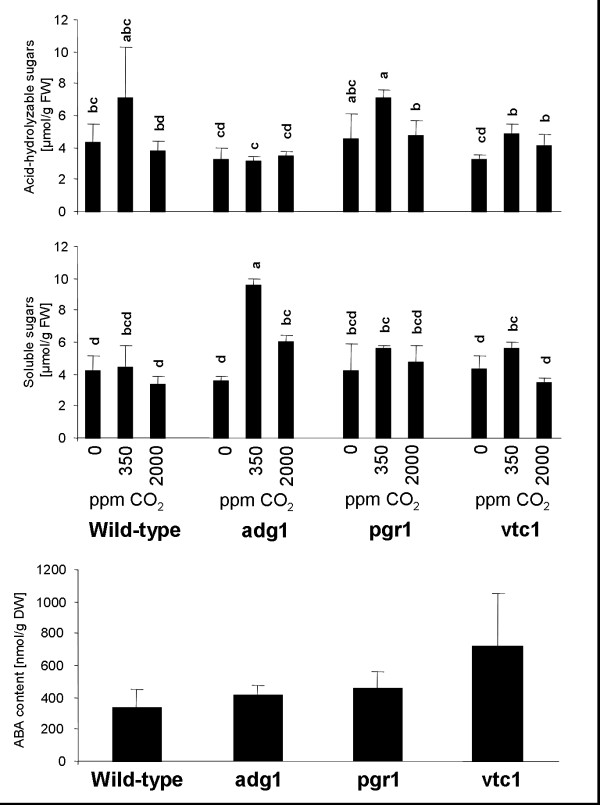
**Content of soluble (A) and hydrolyzable sugars (B) **in *wt, adg1, pgr1 *and *vtc1 *under different CO_2 _regimes (0 ppm, 350 ppm and 2000 ppm CO_2_). The data are means of n = 4–6, ± SE from at least 4 different experiments. **Abscisic acid (ABA) content (C) **in *wt, adg1, pgr1 *and *vtc1 *after fumigation with 350 ppm CO_2_. The data are means of n = 3 ± SE.

In *wt*, *pgr1 *and *vtc1 *the CO_2 _availability barely changed the availability of soluble sugars, while in *adg1*, due to the limitation in chloroplast starch biosynthesis, the sugar concentration was higher in 350 ppm CO_2 _than in *wt*. In parallel, hydrolysable sugars were generally very low in *adg1 *and did not increase in high CO_2_. *pgr1 *and *vtc1 *had similar and slightly higher soluble sugar levels than *wt *at all CO_2 _concentrations applied. In contrast, the concentration of hydrolysable sugars was less in *vtc1 *compared to *pgr1*, which may reflect the effect of limited GDP-mannose pyrophosphorylase activity on cell wall biosynthesis [27]. Generally, in *wt*, *vtc1 *and *pgr1 *the sugar levels were only elevated at ambient CO_2_, but not in saturating CO_2 _demonstrating that in high CO_2_, in which Calvin-cycle activity should be stimulated, carbon assimilation was limited or carbohydrate consumption activated leading to similar carbohydrate pool sizes as in 0 ppm CO_2 _(Figure 2A and 2B).

### ABA levels in ambient air

Consistent with previous observations [14], the ABA content was increased 2.1-fold in *vtc1 *compared to *wt*. *adg1 *and *pgr1 *showed insignificant increases in the range of 1.2- to 1.3-fold, respectively (Figure 2C).

### Photosynthetic performance of wild-type and mutants

To test *wt *and mutants for limitations in photosynthetic electron transport, in the final 2 hours of the fumigation period the photosynthetic response of the plants to a 3.6-increase in the light intensity from 80 to 285 μmol quanta m^-2 ^s^-1 ^was tested by monitoring chlorophyll-a-fluorescence parameters (Table 2). In ambient air, the quantum yield of photosystem II (ΦPSII, (F_M'_-F_S_)/F_M'_) was similar at standard light conditions in *wt*, *adg1 *and *vtc1 *and slightly higher in *pgr1*. In response to increased light, it decreased most in *pgr1 *with a steady-state level of 0.31. In *vtc1*, the steady-state ΦPSII was highest, while in *wt *and *adg1 *it was slightly lower.

In 0 ppm, ΦPSII strongly decreased in all plants prior to the increase in light intensity (Table 2). In response to the 3-fold increase in light intensity ΦPSII further decreased to levels between 0.032 (*adg1*) and 0.093 (*wt*) indicating severe reduction of photosynthetic electron transport efficiency.

At 2000 ppm CO_2 _(Table 2), ΦPSII of all lines was highest at standard light conditions (F_V_/F_M _between 0.663 and 0.734) and dropped in response to increased light to levels around 0.5 in *wt*, *adg1 *and *vtc1 *and as low as 0.35 in *pgr1*.

### Transcript level regulation in response to high and low CO_2_

**Figure 3:**
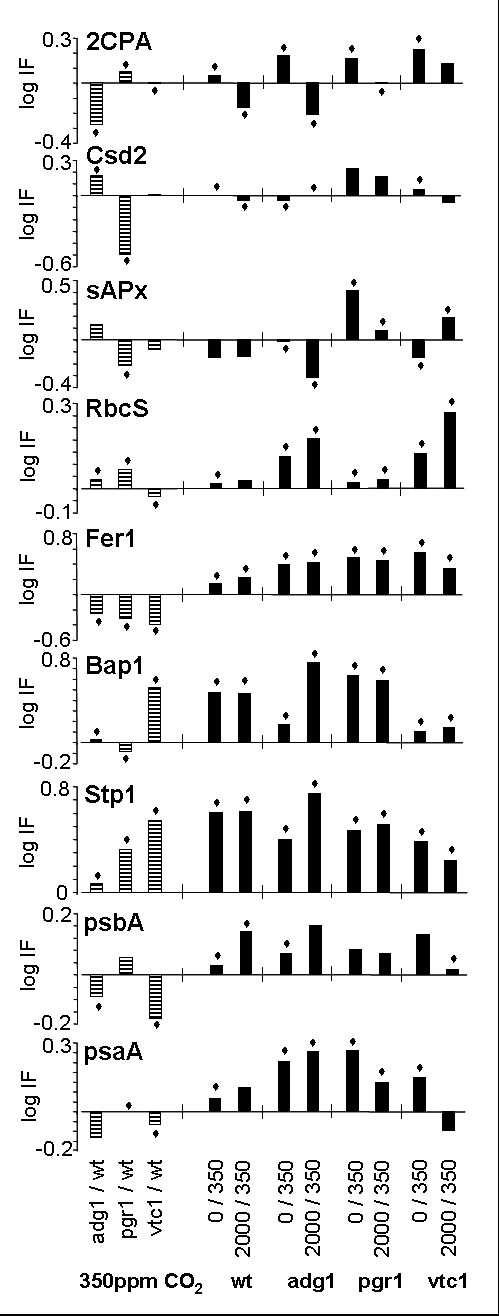
**Semi-quantitative RT-PCR data of selected plastome and nuclear encoded genes**. The PCR products were separated on ethidium bromide containing agarose gels and documented electronically in UV light and analyzed densitometrically. The values are given as the logarithm of the induction factor which is calculated from the ratio of transcript level of WT and mutants at ambient 350 CO_2 _(hatched bars). The black bars represent logarithmic values calculated after fumigation with 0 or 2000 ppm CO_2_, respectively, in relation to corresponding control at 350 ppm CO_2 _for *wt, adg1, pgr1 *and *vtc1*. The data are means of n = 3, (◆) indicates that the SD was less than 30% of the mean value.

Semi-quantitative RT-PCR analyses at least in triplicates were performed to assess the transcript regulation of selected genes in leaves 6 h after fumigation beginning with the respective CO_2 _concentration (Fig. 3). Compared to *wt*, in 350 ppm CO_2 _the transcript levels of the plastidic genes *psaA *and *psbA*, which encode core subunits of photoreaction centre I and II, respectively, and that of the nuclear encoded small subunit of RuBisCO were not significantly changed in the mutants *adg1*, *pgr1 *and *vtc1*, reflecting acclimation. The transcript level for ferritin-1 which has been suggested as marker gene for hydrogen peroxide [28] decreased in all mutants. In contrast, transcripts for Bap1, which is a marker gene for singlet oxygen signalling [28], were specifically increased in the ascorbate deficient mutant *vtc1*.

The transcript levels of the three tested nuclear encoded chloroplast antioxidant enzymes Csd2, sAPx and 2CPA showed mutant specific patterns. Csd2 and sAPx transcript levels were *wt*-like in *vtc1*, increased in *adg1 *and decreased in *pgr1*, while the 2CPA transcript levels were specifically decreased in *adg1*. Stp1 transcript amounts, which are suppressed by the cellular carbohydrate availability [29], were doubled in *pgr1 *and increased 3.5-fold in *vtc1*, but were unchanged in *adg1*.

In *adg1 *and *pgr1*, like in *wt*, the transcript levels of the two plastome encoded genes *psaA *and *psaB *and the nuclear encoded RbcS, Stp1, Bap1 and Ferritin-1 increased in response to 0 and 2000 ppm. Fig. 3 gives the transcript modifications in response to depleting or saturating CO_2 _of wt and mutants each normalized to the respective level in 350 ppm. In *vtc1*, the response of Bap1, which was already strongly increased at 350 ppm, was weakest. In 2000 ppm CO_2 _treated *vtc1 *psbA transcripts were hardly increased and *psaA *transcripts were even slightly decreased indicating a specific response pattern of *vtc1 *to high CO_2_.

Again the three transcripts for nuclear encoded chloroplast antioxidant enzymes Csd2, sAPx and 2CPA showed specific response patterns. Csd2 hardly responded to changes in the CO_2_-availability. Only in *pgr1*, where the transcript levels were decreased at 350 ppm CO_2_, an increase in 0 and 2000 ppm CO_2 _was observed. sAPx transcripts, which were decreased in wt Arabidopsis in response to increased and also decreased CO_2 _availability, strongly decreased in *adg1 *treated with 2000 ppm CO_2 _and increased in *pgr1 *at 0 ppm CO_2_. In *vtc1*, the transcript level responded *wt*-like in 0 ppm CO_2 _and atypically increased in 2000 ppm CO_2_. 2CPA transcripts showed the previously reported CO_2 _dependency [30]. The regulation amplitude increased in *adg1*. The high CO_2_-response was abolished in *pgr1 *and reversed to an increased transcript level in 2000 ppm CO_2 _in *vtc1*.

## Discussion

Photosynthetic activity and metabolism depend on the stoichiometrical assembly and regulated interaction of nuclear and chloroplast encoded proteins [31]. In addition, changing environmental conditions are continuously sensed and used to adjust the photosynthetic apparatus for balanced supply of energy, reductive power and assimilates. The basic mechanism of regulation involves coordination of gene expression. Although various studies on this topic have identified candidate signals, the complexity of interaction and the multiplicity of signals are far from being understood. Here, a set of biochemical and physiological data and the transcripts were analyzed upon variation of the CO_2_-availability in order to tentatively identify potential signalling dependencies (Table 3). Approaching CO_2 _saturation releases the electron pressure within the photosynthetic electron transport chain, increases the acceptor availability at PSI and decreases photorespiration intensity. High quantum yields of PSII (Table 2) indicated efficient electron consumption. Despite metabolic activation, the cellular reduction state of NADP(H) increased in *wt *under saturating CO_2 _(Table 1). In parallel, the ATP availability decreased (Table 1) [6].

### Regulation of ROS-responsive genes

Six hours of illumination in low, ambient or high CO_2 _elicited significant changes in transcript abundance. The increase of ferritin-1 and Bap1 transcript amounts in 0 and 2000 ppm CO_2 _indicates redox imbalances and stimulation of ROS signalling in high as well as low CO_2 _[28]. This is surprising since relaxation of electron pressure should be maximal at saturating CO_2 _with concomitantly low rates of ROS generation. However, a high activation state of the Calvin cycle needed for efficient carbon fixation at saturating CO_2 _depends on a highly reduced thioredoxin system that in turn activates the redox regulated enzymes [32]. The increase of ferritin-1 and Bap-1 transcript levels suggests that regulated electron drainage maintains sufficient electron pressure and is involved in the up-regulation of the ROS-related marker genes.

The CO_2 _dependent response was altered in the mutant genetic backgrounds. In *adg1*, the Bap1 transcript levels were less induced in 0 ppm and more in 2000 ppm (Figure 3) demonstrating that limitations in chloroplast carbohydrate storage affect the responsiveness of singlet oxygen-signalling. It is tempting to assume that in *adg1 *increased APx and SOD activities (Figure 1) antagonized Bap1 induction in low CO_2 _(Figure 3) due to higher antioxidant protection, while the high transcript accumulation in 2000 ppm CO_2 _results from carbohydrate inhibition of photosynthetic electron transport due to insufficient capacities for starch biosynthesis. Transcript amount co-regulation analysis shows among all genes tested the strongest correlation of Bap1 with the ascorbate content (K = -0.74; Table 3). Consistent with the hypothesis by op den Camp et al. [28] that Bap1 is regulated by singlet oxygen, the antagonistic effect of ascorbate availability and the negative correlation with the activity of antioxidant enzymes supports the conclusion that the overall cellular antioxidant capacity controls Bap1 induction. Because the total glutathione content was significantly decreased in *adg1 *in 350 ppm at the begin and end of the fumigation time (Figure 1A), a special regulatory function in Bap1-regulation is indicated for the ascorbate-specific components of the Halliwell-Asada-Cycle [33].

In contrast, for ferritin-1, which is supposed to be induced specifically by H_2_O_2 _[28], no such strict correlation was observed with the availability of low molecular weight antioxidants or the activity of antioxidant enzymes. Highest correlation was observed with the availability of hydrolysable sugars (K = -0.62; Table 3) suggesting regulation by carbohydrate metabolism. A similar negative correlation with the contents of hydrolysable sugars was observed for Stp1, which encodes a plasma-membrane monosaccharide transporter [29]. Even stronger than for ferritin-1 transcript abundance and Bap1 transcript abundance, for Stp1 a positive correlation with the glutathione content and the redox state of the glutathione pool was observed (Table 3) suggesting glutathione-dependent regulation. However, excluding *adg1*-data from the correlation analysis demonstrated that at least for Bap1 and Stp1 the high correlation with glutathione data results from the *adg1*-specific regulation of the glutathione pool and, therefore, may also result from disturbed chloroplast carbohydrate metabolism.

### Ascorbate-dependent regulation

In a genome-wide transcript analysis of *vtc1 *plants, Pastori et al. [14] identified 171 genes with altered expression, among which defence genes constituted a significant subgroup. Here, in the steady state, in 350 ppm CO_2_, to which the plants were adapted to, the transcript levels of 2CPA, Csd2 and sAPx were balanced. The transcript level of ferritin-1 was even decreased. The transcript level of Bap-1 was 3.73-fold increased and constitutively high upon variation of the CO_2_-availability, demonstrating that the transcript abundance is dominantly regulated by the mutational defect in ascorbate biosynthesis.

The transcript levels of Bap1, Fer1, RbcS and Stp1, although to different extends, negatively correlated with the ascorbate content and positively with the reduction state. Apparently metabolically induced alterations in ascorbate levels caused similar transcriptional changes as genetic mutations in ascorbate biosynthesis [14]. In our analysis the correlation between ascorbate and defence gene expression was stronger when the data set of the *vtc1*-mutant was not included (K = -0.67) demonstrating that in *vtc1 *hardening responses may mask signalling induced by the variation of CO_2_-availability.

The strongest relationship between the reduction state of ascorbate and transcript level regulation was observed for 2CPA. In previous experiments strong suppression of 2CPA transcription was observed upon ascorbate application [29,34,35]. Here, responses upon internal variation of the ascorbate content were analyzed. A strong negative correlation (-86 %; Table 3) with the reduction state of ascorbate, but absence of correlation with the ascorbate content (K = 0.23; Table 3) excludes sensing the ascorbate availability. It is postulated that either specifically dehydroascorbate or, more likely, the electron consumption in dehydroascorbate reduction regulates 2CPA transcript abundance.

### Coupling of transcript abundance regulation

A set of four genes, i.e. Bap1, psaA, psbA and Stp1, showed a similar transcript pattern in response to the seven metabolic parameters, i.e. glutathione contents and reduction state, 3-PGA and DHAP contents, the calculated NADPH reduction state and assimilatory force, and the hydrolysable sugars (Table 3). It should be noted that transcript levels of psaA and psbA changed in parallel in response to the imposed metabolic and mutational strains. This contrasts the anti-parallel responses of psaAB and psbB observed upon transfer to photosystem I and II-specific light regime previously described by Pfannschmidt et al. [11]. It is concluded that a variation between 0 and 2000 ppm CO_2 _in the mutant background elicits more severe changes in metabolism and signalling than altering the light quality from red to far red at very low photon flux density [11]. However, the photosystem I transcript psaA decreased relative to the photosystem II transcript psbA when the electron pressure was reduced by increasing the CO_2_-availability (Figure 3), e.g. from 1.07 (0 ppm) to 1 (350 ppm) to 0.88 (2000 ppm) in *wt *indicating a stronger transcription of genes for the PS-I reaction centre protein in 0 ppm CO_2 _and those encoding PS-II reaction centre proteins in 2000 ppm CO_2_. In *adg1*, the gradual response was unaffected, however generally the transcript abundance of psbA was higher than that of psaA (Figure 3). In *pgr1 *the relative transcript abundance normalized to *wt *at 350 ppm was also higher under all three CO_2 _conditions, suggesting that the higher psbA transcript levels were caused by photoinhibitory high carbohydrate availability or a limitation in the Rieske activity.

It is tempting to suggest that the signal is transmitted by PQ-dependent redox signals. However, in *vtc1 *more *psaA *than *psbA *was observed at 350 ppm CO_2_, a balanced psaA/psbA ratio in 0 ppm CO_2 _and an inverted ratio in 2000 ppm, demonstrating that the ascorbate availability affects the stoichiometry of transcripts for the photoreaction centres upon variation of the CO_2_-availability. After 6 h fumigation with 350 ppm CO_2_, the steady state quantum yield of PS-II ((F_M'_-F_S_)/F_M'_) was *wt*-like (Table 2), while the quantum yield was adjusted to 1.23-fold higher levels within 4.5 min of illumination upon doubling of the light intensity (Table 2). On the background of decreased ascorbate availability (Figure 2A) this demonstrates that the photosynthetic electron transport chain was otherwise protected. Chlorophyll fluorescence analysis showed that the acclimation was insufficient to protect the photosynthetic membrane in low CO_2_. The low quantum yield of PS-II (Table 2) demonstrates even stronger photosynthetic impairment than in *wt*. That the regulation of the quantum yield of PS-II does not correlate with the psaA/psbA-ratios, supports the hypothesis that the regulation of the transcript abundance of the photoreaction centre proteins is more dependent on ascorbate-specific signals than on the redox state of the PQ-pool. The PQ-dependent long term acclimation response postulated by Pfannschmidt et al. [11] appears further subordinate to signals linked to the metabolic state of the PGA and DHAP concentrations, the NADPH/NADP^+ ^ratio, the assimilatory force and hydrolysable sugars (Table 3). In *adg1 *a perfect positive correlation (K = 1) was observed between the concentration of soluble sugars and the psbA transcript levels, whereas the other lines showed a high negative correlation (*wt *: K = -1;*pgr1 *: K = -0.96;*vtc1*: K = -1), highlighting the importance of carbohydrate-dependent signals in psaA and psbA regulation.

### Correlation with the adenylate status

In animals, the energy status sensed for instance via insulin-like growth factor 1 or by AMP-dependent kinases plays an important role in regulation of gene expression [36]. However, here except an only weak (K = 0.41) correlation for 2CPA, no correlations between the adenylate status and transcript levels were indicated. Due to the photoautotrophic nature of plants, plants rarely encountered energy deprivation. Further on, the chloroplast and cellular adenylate status directly coordinates metabolic pathways via feed-back and feed-forward mechanisms [37]. The lack of strong energy-linked regulation demonstrates that the adenylate phosphorylation state may not be a major signal, which is directly linked to the regulation of nuclear gene expression in context of photosynthesis.

However, the analysis of *pgr1 *showed a mutant specific regulation of Csd2, sAPx and 2CPA upon altered CO_2_-availability (Figure 3). Because *pgr1 *is unable to acidify the thylakoid lumen below pH 6 due to a mutation in the Rieske protein [18], the ATP/ADP ratio was very low in the morning and generally decreased irrespective of the CO_2_concentration (Table 1). The concentrations of most metabolites were not significantly changed in response to altered CO_2 _availability compared to *wt *indicating efficient metabolic compensation (Figure 3). The transcript levels of Csd2, sAPx and 2CPA were regulated in a mutant specific manner, while RbcS, Bap1, Fer1 and Stp1 responded *wt*-like demonstrating that ROS- and carbohydrate signalling pathways were unaffected [28,29,38]. Upon the different treatments of *pgr1 *the transcript abundance of sAPx highly correlated with the calculated reduction state of NADPH (K = 0.99), while no correlation was observed in the other plant lines. It is therefore concluded that the mutation in the Rieske protein limits the fine-control of sAPx expression and makes it more dependent on stromal redox signals. The difference in sAPx regulation between *pgr1 *and *wt *also demonstrates that in *wt *the Rieske protein influences sAPx expression. Excluding ROS-signalling, because the Bap1 control was *wt*-like, either the redox state of the PQ pool or thylakoid acidification/the adenylate status may modulate sAPx transcript abundance.

The Csd2 transcript levels positively correlated with APx activity in all lines under all treatments (Table 3). Concomitantly, in *pgr1*, also an almost perfect correlation was observed with the redox state of NADPH. Compared to sAPx the Csd2 transcript level was regulated with higher amplitudes. sAPx and Csd2 transcripts encode two prominent chloroplast antioxidant enzymes, which successively act in superoxide and H_2_O_2 _detoxification in the stromal part of the Halliwell-Asada cycle (ascorbate-dependent water-water cycle) [33]. Coordinated regulation of transcript abundance points out expressional co-regulation.

## Conclusion

In the cell, metabolite, redox and energy signals are tightly linked, which makes differentiation of signalling cascades difficult. This study demonstrates that comparison of mutants with specific limitations in the coordination of plant acclimation at least transiently uncouples signalling branches. Comparison of *adg1*, *pgr1 *and *vtc1 *with Arabidopsis wild-type plants showed coordinated expression of Bap1, psaA, psaA and Stp1, which have been discussed previously to respond specifically to singlet oxygen [28], the redox state of the PQ pool [11] and monosaccharide availability [29], respectively. Like Ferritin-1, Bap1 and Stp1 correlated strongest with the ascorbate contents, while for psaA and psbA a stronger link to the assimilatory force and NADPH/NADP^+ ^ratio was indicated. Correlation of 2CPA expression, whose transcription is controlled by the acceptor availability at photosystem I in *wt *[30], with the redox state of the ascorbate pool further strengthens the links between the antioxidant system and the photosynthetic electron transport and more specifically chloroplast-to-nucleus signalling. It is postulated that during evolution a stabilized network has evolved which links photosynthetic metabolism to nuclear gene expression. Mutants might be well balanced under standard conditions, but application of environmental changes leads to an altered acclimation in comparison to *wt*, which allows to tentatively differentiate parallel induced signalling cascades.

## Methods

### Plant material and growth conditions

*Arabidopsis thaliana wt *(Col-0), the mutants *adg1 *[16], *pgr1 *[15] and *vtc1 *[17] grew in controlled environment (10 h of light, 100 μmol quanta m^-2 ^s^-1^, 23°C and 14 h darkness at 18°C; 50% relative humidity) on a 1:1:1 mixture of Frühsdorfer Erde Klocke P, perlite and vermiculite for 5 weeks. Beginning 2 h after onset of light, sets of plants (*wt*, *adg1*, *pgr1 *and *vtc1*) were fumigated for 6 h with synthetic air in 28 l Perspex chambers or 5-l Sekuroka^® ^glove bags with 0 and 2000 ppm CO_2_, respectively, or maintained in ambient air. A Millipore Tylan RO 7030 system controlled the gas flow at 2 l/min.

### Metabolite analysis

Total and reduced ascorbate was quantified spectrophotometrically according to [39] from plant material frozen in liquid nitrogen by recording the decrease of absorption at 265 nm following addition of ascorbate oxidase. Glutathione contents were determined fluorimetrically after derivatization with monobromobimane and HPLC separation on a "reverse phase" Hypersil BDS-C15 5 μm column from tissue extracted in 0.1 M HCl and 5 mM diethylenetriamine pentaacetic acid [40]. 3-PGA and DHAP contents of perchloric acid extracts were quantified according to Dietz & Heber [41], ATP and ADP with firefly enzyme according to the luminometric method described by Kaiser & Urbach [42]. Assimilatory force and NADPH-reduction state were calculated as described in Dietz & Heber [24]. Reducible hydrolysable and soluble sugars were determined according to Yemm & Willis [43] with anthrone reagent. ABA contents were quantified according to Weiler [44] from freeze-dried leaf material.

### Chlorophyll-a-fluorescence measurements

Between 4–6 h after onset of the CO_2 _fumigation, the response of the mutants to an increase in light intensity to 285 μmol quanta m^-2 ^s^-1 ^was monitored for 4 min using a Mini-PAM Fluorometer (Walz, Effeltrich, Germany). 30 s before and during the illumination with 285 μmol quanta m^-2 ^s^-1^, the fluorescence parameters F_S _and F_M' _[45] were determined every 30 s with saturating light pulses (1s; >3000 μmol quanta m^-2 ^s^-1^). The quantum yield of PS-II (ΦPSII) was calculated as (F_M' _- F_S_)/F_M'_.

### Determination of protein contents and biochemical analyses of enzyme activities

Enzyme activities were determined according to [34] and standardized on protein contents of the samples determined with the BIORAD protein assay (BioRad Laboratories, München, Germany).

### RNA isolation, cDNA synthesis and RT-PCR analysis

Approximately 100 mg plant material pulverized in liquid nitrogen was extracted in 500 μl 100 mM Tris-HCl, pH 8.5, 25 mM EDTA, 25 mM EGTA, 100 mM β-mercaptoethanol and 2% SDS with 500 μl phenol and 300 μl chloroform. The aqueous phase was re-extracted first with 1 ml phenol/chloroform (1:1), then with 1 ml chloroform prior to precipitation of the RNA at 4°C by addition of 1 ml isopropanol. For further purification the precipitate was dissolved in 10 mM Tris, 1 mM EDTA, re-precipitated with 3 volumes 8 M LiCl and washed with 70 % ethanol. The RNA dissolved in DEPC-treated water was quantified spectrophotometrically. Residual DNA was removed by treatment with RNase-free DNase (1 U; Promega). Prior to cDNA synthesis, the DNase was inactivated by adding EDTA to a final concentration of 2.5 mM and by incubation at 70°C for 20 min. 5 μg DNase treated RNA was used for cDNA synthesis using oligo(dT) and a primer mix containing a mixture of oligonucleotides matching all plastid-encoded genes as primers [46] for 1 h at 42°C. The samples were standardized on actin-2 transcript amount. Genes of interest were analysed by semi-quantitative RT-PCR (PCR settings: 1 cycle at 94°C for 3 min, for the optimized number of cycles: 45 s 94°C, 30 s 50°C, and 15 s 72°C). Following separation of the PCR products on ethidiumbromide stained agarose gels, the bands were quantified densitometrically [34]. Each band was normalized against the intensity obtained with the same cDNA using the actin-specific primers. The primers and gene code numbers were as follows: *actin *(At5g09810) sense 5'-TACAACGAGCTTCGTGTTGC-3', antisense 5'-GGACAACGGAATCTCTCAGC-3'; *psbA *(AtCg00020) sense 5'-GAAAATCAATCGGCCAAAAT-3', antisense 5'-TTACCCAATCTGGGAAGCTG-3'; *psaA *(AtCg00350) sense 5'-GATCTAATCCGCCACGAAAA-3', antisense 5'-CAGGTGGTTTGGCCAATAGT-3'; *RbcS *(all isoforms) sense 5'-AGAAGTAATGGCTTCCTC-3', antisense 5'-AAGCTTCGGTGAAGCTTG-3'; *Csd2 *(At2g28190) sense 5'-CTCCGTTCCTCTTTCAGC-3', antisense 5'-GCGTCAAGCCAATCACAC-3'; *2CPA *(At3g11630) sense 5'-CTCTCCATCTGTTTCTTT-3', antisense 5'-GTACCTTTTTCGTATCAT-3'; *sAPx *(At4g08390) sense 5'-TGTTCCAGTTAGCTAGTG-3', antisense 5'-GGTTGAGTAAATTAGGTGC-3'; *Fer1 *(At5g01600) sense 5'-ATGGCCTCAAACGCACTCT-3', antisense 5'-CTAGTCCCTTCATAGCAACG-3', *Bap1 *(At3g61190) sense 5'-CTAAACCGGAGACCCATC-3', antisense 5'-AGTGACCTTCAGGTGAATAC-3'; *Stp1*(AT1g11260) sense 5'-TTCTTTCAACAGCTAACCGGA-3', antisense 5'-GGCTAATACACTTTTTCCTTTA-3'.

### Statistical analysis

From the calculated means and standard deviations the significance of differences was determined by Student's *t *test. Data sets were designated significantly different, if the *P *value was below 0.05. Coefficiency analysis was performed by Pearson correlation analysis. Coefficients higher than r = | 0.6 | was defined as strong correlation, and between | 0.3 | and | 0.6 | as weak correlations.

## Abbreviations

2CP: 2-cysteine peroxiredoxin; 3-PGA: 3-phosphoglycerate; APx: ascorbate peroxidase; cDNA: copy DNA; Csd: cupper/zinc superoxide dismutase; DEPC: diethyl pyrocarbonate; DHAP: dihydroxyacetone phosphate; F_A_: assimilatory force; FW: fresh weight; PQ: plastoquinone; Prx: peroxiredoxin; PS: photosystem; ROS: reactive oxygen species; RT-PCR: amplification of transcripts by polymerase chain reaction after reverse transcription into cDNA; RuBisCO: ribulose-1,5-bisphosphate carboxylase/oxygenase; SOD: superoxide dismutase; *wt*: wild-type

## Authors' contributions

DW carried out the metabolite analyses, most of the gene expression analyses, performed the statistical analysis and contributed to writing the manuscript. MB performed preparatory work for the gene expression analysis, carried out the chlorophyll-a fluorescence analysis, was involved in designing and validation of the project and contributed to writing of the manuscript. AK was involved in transcript analysis and project development, and helped with the manuscript, RS provided technical support and discussion, WH determined the ABA-contents and K-JD was the principal investigator who coordinated the project and was involved in data interpretation and writing of the manuscript. All authors agreed on the final manuscript.

## Figures and Tables

**Table 1:** Energetisation and phosphorylation state under influence of different acceptor availabilities: ATP content, ADP content, 3-PGA content and DHAP content in *wt, adg1, pgr1 *and *vtc1 *under different CO_2 _regimes (0 ppm, 350 ppm and 2000 ppm CO_2_).

			**Start**		**0 ppm CO_**2**_**		**350 ppm CO_**2**_**		**2000 ppm CO_**2**_**	
**wt**	ATP	[nmol/g FW]	97.6 ± 15.6	c	172.9 ± 31.9	a	79.1 ± 14.7	eg	60.9 ± 9.5	efg
	ADP		17.5 ± 2.8	c	29.7 ± 4.5	b	36.2 ± 10.4	b	33.1 ± 23.3	bc
	3PGA		294.0 ± 149.2	abc	117.0 ± 73.3	c	404.1 ± 44.4	b	301.0 ± 126.4	bc
	DHAP		100.0 ± 10.4	abc	71.7 ± 40.2	c	36.1 ± 15.3	b	76.4 ± 49.5	bc
	
	ATP/ADP		5.6		5.8		2.2		1.8	
	[NADPH/NADP^+^]_calc_		23.0		34.0		16.7		40.3	
	Assimilatory Force		333.0		600.4		87.6		248.7	

**adg1**	ATP	[nmol/g FW]	68.2 ± 8.7	e	149.6 ± 25.6	abc	88.8 ± 5.1	c	77.9 ± 13.4	ed
	ADP		20.6 ± 10.1	bc	113.6 ± 25.0	a	39.7 ± 24.5	bc	18.8 ± 4.2	bc
	3PGA		341.2 ± 123.3	b	113.7 ± 37.5	c	487.6 ± 81.3	ab	256.4 ± 123.3	bc
	DHAP		46.0 ± 15.0	b	54.0 ± 38.8	c	45.3 ± 24.2	ab	75.8 ± 96.2	bc
	
	ATP/ADP		3.3		1.3		2.2		4.2	
	[NADPH/NADP^+^]_calc_		16.6		63.9		16.9		25.8	
	Assimilatory Force		132.1		456.7		91.0		289.5	

**pgr1**	ATP	[nmol/g FW]	71.1 ± 12.4	ef	57.5 ± 9.3	ef	84.6 ± 4.4	d	53.4 ± 9.3	f
	ADP		24.5 ± 10.3	bc	29.5 ± 7.4	b	44.3 ± 12.7	b	24.2 ± 11.0	c
	3PGA		511.9 ± 142.0	ab	137.2 ± 49.2	c	522.5 ± 14.3	a	249.9 ± 96.8	bc
	DHAP		90.0 ± 13.0	ab	43.5 ± 27.3	c	46.1 ± 11.8	a	44.1 ± 13.4	bc
	
	ATP/ADP		2.9		1.9		1.9		2.2	
	[NADPH/NADP^+^]_calc_		22.9		44.4		18.4		28.2	
	Assimilatory Force		172.3		311.0		86.5		173.0	

**vtc1**	ATP	[nmol/g FW]	116.0 ± 21.6	bc	101.6 ± 38.0	bcdg	96.8 ± 9.8	cdg	79.9 ± 12.7	ceg
	ADP		11.9 ± 4.5	bc	74.8 ± 10.3	a	33.1 ± 14.1	b	39.2 ± 22.0	bc
	3PGA		355.1 ± 35.9	ab	144.3 ± 96.5	c	392.2 ± 130.9	b	288.3 ± 61.1	bc
	DHAP		40.0 ± 15.0	ab	56.4 ± 23.2	c	50.9 ± 24.3	b	29.0 ± 9.8	bc
	
	ATP/ADP		9.8		1.4		2.9		2.0	
	[NADPH/NADP^+^]_calc_		5.3		58.5		17.9		19.4	
	Assimilatory Force		110.4		383.4		127.3		98.5	

Different letters indicate significant differences. The data are means of n = 4–6, ± SE from at least 4 different experiments. ATP/ADP ratio, [NADPH]/[NADP^+^]_calc _and assimilatory force F_A _(according to Dietz & Heber 1989). These derived parameters were calculated from the primary data above.

**Table 2:** Effective quantum yield of PSII at different CO_2 _regimes (0 ppm, 350 ppm and 2000 ppm CO_2_) in response to an increase in the light intensity from 80 to 285 μmol quanta m^-2^s^-1^.

			wt	adg1	pgr1	vtc1
0 ppm CO_2_		0 min	0.318 ± 0.071	0.167 ± 0.050	0.192 ± 0.090	0.278 ± 0.063
		4.5 min	0.093 ± 0.040	0.032 ± 0.008	0.071 ± 0.043	0.051 ± 0.039

350 ppm CO_2_		0 min	0.580 ± 0.039	0.596 ± 0.029	0.661 ± 0.033	0.599 ± 0.041
		4.5 min	0.388 ± 0.036	0.395 ± 0.029	0.314 ± 0.037	0.437 ± 0.032

2000 ppm CO_2_		0 min	0.663 ± 0.025	0.704 ± 0.022	0.734 ± 0.012	0.710 ± 0.019
		4.5 min	0.518 ± 0.033	0.482 ± 0.046	0.345 ± 0.026	0.519 ± 0.037

The data are means of 6 measurements.

**Table 3:** Correlation analysis of metabolite data, enzyme activities and transcript levels.

	psaA	psbA	2CPA	sAPx	Csd2	RbcS	Bap1	Fer	Stp1
PSII [0 min]	- 0.28	- 0.08	- 0.34*	- 0.22	- 0.17	+ 0.07	- 0.12	+ 0.08	- 0.11
PS II [4.5 min]	- 0.38*	- 0.14	- 0.33*	+ 0.03	- 0.03	+ 0.07	- 0.09	+ 0.24	- 0.15
ATP	- 0.12	- 0.39*	+ 0.58*	+ 0.03	+ 0.35*	- 0.03	- 0.19	- 0.12	- 0.24
ADP	- 0.10	- 0.22	+ 0.09	+ 0.23	+ 0.28	+ 0.18	- 0.20	+ 0.09	- 0.21
ATP/ADP	+ 0.05	- 0.07	+ 0.41*	- 0.31*	+ 0.19	- 0.01	+ 0.14	- 0.19	+ 0.09

Soluble Sugars	- 0.01	+ 0.01	+ 0.02	- 0.35*	- 0.71**	- 0.33*	- 0.25	- 0.34*	- 0.20
Hydrolyzable Sugars	- 0.43*	- 0.46*	+ 0.12	+ 0.07	+ 0.47*	- 0.29	- 0.45*	- 0.62**	- 0.60**
3PGA	- 0.51*	- 0.41*	- 0.07	+ 0.02	- 0.07	- 0.36*	- 0.55*	- 0.32*	- 0.63**
DHAP	+ 0.50*	+ 0.39*	+ 0.35*	- 0.27	+ 0.36*	+ 0.10	+ 0.34*	+ 0.25	+ 0.48*
[NADPH/NADP+]_calc_	+ 0.52*	+ 0.40*	+ 0.09	+ 0.08	+ 0.15	+ 0.23	+ 0.33*	+ 0.32*	+ 0.47*
Assimilatory Force	+ 0.49*	+ 0.29	+ 0.42*	- 0.08	+ 0.24	+ 0.17	+ 0.30*	+ 0.16	+ 0.43*

Ascorbate Content	- 0.08	- 0.04	+ 0.23	+ 0.29	+ 0.02	- 0.41*	- 0.74**	- 0.32*	- 0.46*
Ascorbate %red	- 0.10	+ 0.16	- 0.86**	- 0.25	+ 0.01	+ 0.47*	+ 0.50*	+ 0.30*	+ 0.43*
GSH Content	+ 0.61**	+ 0.64**	- 0.14	+ 0.02	- 0.67**	- 0.13	+ 0.54*	+ 0.46*	+ 0.67**
GSH % red	+ 0.41*	+ 0.37*	- 0.02	- 0.08	- 0.46*	+ 0.09	+ 0.46*	+ 0.35*	+ 0.60**

CAT Activity	- 0.13	- 0.11	+ 0.22	+ 0.37*	- 0.11	- 0.46*	- 0.44*	+ 0.10	- 0.24
SOD Activity	- 0.56*	- 0.41*	- 0.21	- 0.12	+ 0.80**	+ 0.46*	- 0.27	- 0.38*	- 0.51*
GR Activity	- 0.33*	- 0.23	- 0.11	+ 0.20	- 0.38*	- 0.56*	- 0.24	- 0.27	- 0.34*
total APx Activity	- 0.59*	- 0.44*	- 0.39*	-0.10	+ 0.70**	+ 0.60 **	- 0.24	- 0.31*	- 0.45*

The table shows the correlation coefficient K calculated between metabolite, enzyme activities and every single transcript detected by semi-quantitative RT-PCR. Bold printed numbers with '*' stand for 0.3<|K| <0.6 and bold printed numbers with ' ** ' for strong negative and positive correlations 0.6 = |K|.
